# A survey of knowledge, attitudes, barriers and support needs in providing hepatitis B care among GPs practising in Australia

**DOI:** 10.1186/s12875-022-01754-3

**Published:** 2022-06-02

**Authors:** Yinzong Xiao, Caroline van Gemert, Jess Howell, Jack Wallace, Jacqueline Richmond, Emily Adamson, Alexander Thompson, Margaret Hellard

**Affiliations:** 1 Burnet Institute, Melbourne, VIC 3004 Australia; 2 Department of Gastroenterology, St Vincent’s Hospital, Fitzroy, VIC 3065 Australia; 3 University of Melbourne, Parkville, VIC 3010 Australia; 4 La Trobe University, Bundoora, VIC 3086 Australia; 5 Centre for Social Research in Health, UNSW Australia, Kensington, NSW 2052 Australia; 6 School of Public Health and Preventive Medicine, Monash University, Melbourne, VIC 3004 Australia; 7 Department of Infectious Diseases, The Alfred and Monash University, Melbourne, VIC 3004 Australia; 8 The Peter Doherty Institute for Infection and Immunity, MelbourneVictoria, 3000 Australia

**Keywords:** Chronic hepatitis B, General practitioner, Primary care

## Abstract

**Background:**

In Australia, only 22% of people with chronic hepatitis B (CHB) are clinically managed; and a national effort is engaging primary care workforce in providing CHB-related care. This study explored CHB-related knowledge, attitudes, barriers and support needs of general practitioners (GPs).

**Methods:**

A survey was sent to a random sample of 1,000 Australian GPs in April- October 2018; 134 of 978 eligible GPs completed the questionnaire (14%).

**Results:**

Respondents had high knowledge of at-risk populations (> 79%) and hepatitis B serology (82%), and most saw hepatitis B testing and monitoring as part of their work (95% and 86%, respectively). However, the survey revealed low knowledge, awareness and intention with respect to hepatitis B treatment: 23% correctly understood treatment initiation; 40% were aware that treatment for CHB could be dispensed in the community; 23% agreed that prescribing was part of their work. Lack of time was considered the greatest barrier (38%) and clear guidelines was the most important facilitator to providing care (72%).

**Conclusion:**

Interventions are needed to generate interest and skills to provide CHB-related care by GPs.

**Supplementary Information:**

The online version contains supplementary material available at 10.1186/s12875-022-01754-3.

## Introduction

In Australia in 2019, an estimated 226,566 people were living with chronic hepatitis B (CHB) [1]. Despite free testing and highly subsidised healthcare services for CHB clinical management and treatment, CHB remains underdiagnosed and undertreated in Australia. In 2019, over one-third of people with CHB were not diagnosed, approximately 20% received clinical management, and approximately three-quarters of the 71,544 people estimated to require antiviral therapy were not treated [2–4].

Increasing the number of people diagnosed with CHB is critical. In Australia, hepatitis B testing is mostly general practitioner (GP) initiated. Previously identified challenges contributing to insufficient hepatitis B testing include lack of awareness of at-risk populations, the asymptomatic nature of CHB, and inadequate knowledge and support for providing hepatitis B testing [5–8].

Primary care models of chronic disease management can improve both provider and patient-level outcomes [9, 10]. CHB is now considered a condition suitable to be managed in primary care given the antiviral drugs are effective, safe and cheap, with the availability of a simplified treatment algorithm [11]. Since July 2015, accredited GPs (S100 prescribers) have been able to prescribe CHB treatment in Australia, with medication available through community and hospital pharmacies [12]. However, specialists continue to perform most CHB clinical management; in 2018, GPs were responsible for only 11% of treatment prescriptions [1, 13].

Previous studies highlight challenges to providing CHB treatment in primary care, including lack of prescribing rights (prior to the aforementioned regulatory change), knowledge gaps, cultural barriers and competing priorities [14–18]. While critical elements in providing clinical care in primary care settings at an organisational and provider level have been identified [8], there is little data on GPs’ attitudes, structural barriers, and support needs with respect to providing CHB-related care.

This study explored GPs’ knowledge, confidence and attitudes to delivering CHB-related care and identifed barriers and facilitators in testing, monitoring and treating CHB. The aim was to inform interventions to increase GPs engagement in providing hepatitis B testing, clinical management and treatment in Australia.

## Methods

### Study design

An Australia wide, cross-sectional survey was conducted in April- October 2018. A random sample of 1,000 GPs was selected from the national database of the Australian Medical Publishing Company (AMPCo), the largest commercial database of medical professionals in Australia. The sample size was determined to reflect the GP population practising in 2018 with a confidence level of 95%, a margin of error of 10% and a response rate of 10% [19].

All selected GPs were sent a letter of invitation, information about the program, a paper questionnaire with an online link, and a pre-paid envelope. GPs had the option to complete the questionnaire on paper or online. Reminder letters were posted to those who did not respond one and two months after the first mailout, or emailed to those with known email address.

### Data collection

A 21-item questionnaire (Table s1) was developed after a literature review. It was designed to measure participants’ hepatitis B knowledge, professional identity, attitudes and confidence in providing CHB-related care, and identify self-reported barriers to and support needs for CHB practice. The questionnaire was pilot tested with five GPs.

Participants answered questions about demographics (age, gender, language spoken other than English), practice-related characteristics (working hours per week, practice type, current hepatitis B prescriber), country of primary medical degree (Australia/overseas), and patient profile (proportion of their patients born in hepatitis B endemic countries, with a map provided highlighting endemic countries).

To understand GPs’ hepatitis B-related knowledge, participants were asked to select priority groups to be tested for hepatitis B; and to interpret hepatitis B testing results using multiple-choice questions; knowledge of CHB treatment was measured by responses to three statements (Table s1). Participants’ attitudes to providing CHB-related care were measured by identifying their role in screening, monitoring and treating, as well as their confidence in undertaking these tasks. The questionnaire asked about barriers and facilitators to providing CHB-related care, and their intention of becoming a hepatitis B prescriber. Two questions asked about participants’ liver cancer screening practice and resource use in practice, which were not discussed in this paper (Table s3).

### Data analysis

Returned survey data were entered into REDCap 8.5.11 (TN, USA) and analysed using Stata 13 (Texas, USA). Medians and interquartile ranges (IQRs) were used to describe non-normally distributed quantitative data such as age and self-reported confidence score. Proportions were used to describe categorical data. Free-text responses/comments on barriers and support needs were grouped into themes using NVivo 12. For knowledge-related questions, the number of people answering correctly was recorded; answers of ‘don’t know’/ ‘unsure’ were recorded as incorrectly.

The postcodes of participants’ main practices were mapped to states and Primary Health Networks (PHNs) [20, 21]. Demographics of age, gender and practice states were compared between respondents and non-respondents (data exported from AMPCo dataset) using chi square tests.

A “critical appraisal checklist for a questionnaire study” is included in Table s2 [22]. Ethics approval was obtained from the Alfred Hospital Ethics Committee (630/17).

## Results

**Fig. 1:**
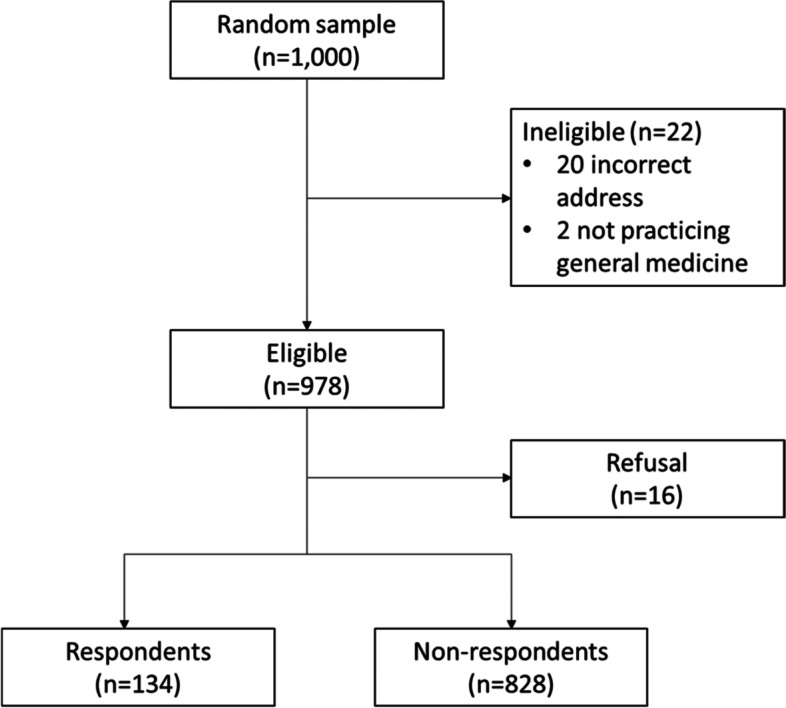
Flow-chart showing participation of Australian GPs in the hepatitis B management survey, 2018

Of 1000 GPs sampled, 978 were eligible, and 134 GPs returned a completed questionnaire (14% response rate) (Fig. 1). Age and gender profile of respondents and non-respondents were similar, however a higher proportion of respondents were from Victoria (*p* = 0.04) (Table 1).

**Table 1:** Comparison of age, gender and practice locations of responding and non-responding GPs

	Respondents (*n* = 134)	Non-respondents (*n* = 828)	*p*-value
Age > 50 (n, %)	85 (64)^a^	477 (57) ^b^	0.20
Female (n, %)	71 (53)	359 (43)	0.05
Practice state (n, %)			
New South Wales	43 (32)	288 (35)	**0.04**
Victoria	40 (30)	173 (21)	
Queensland	20 (15)	180 (22)	
Western Australia	14 (10)	83 (10)	
South Australia	12 (9)	61 (7)	
North Territory	3 (2)	5 (1)	
Australian Capital Territory	2 (1)	15 (2)	
Tasmania	0	23 (3)	
CHB prevalence in practice PHN (%) (mean, SD)	1.00 (0.03)	0.99 (0.01)	0.60
CHB prevalence of practice PHN (%) (n, %)			
[0.52, 0.76)	36 (27)	221 (27)	0.80
[0.76, 1.00)	43 (32)	284 (34)	
[1.00, 1.23)	19 (14)	137 (17)	
[1.23,1.47)	28 (21)	149 (18)	
[1.47, 1.71]	8 (6)	37 (4)	

^a^ Of 133 respondents with age data available. ^b^ Of 823 non-respondents with age data available

*PHN* Primary Health Network

Demographics data of non-respondents were sourced from the AMPCo database. Postcode data of participants’ practice was mapped to states or territories and to PHN as per the 2016–2017 PHN concordance file released by the Australian Bureau of Statistics. The CHB prevalence of each PHN was sourced from Australian hepatitis B mapping project data in 2017

### Description of respondents

Fifty-three percent of respondents were female, with a median age of 56 years (IQR 47–64) (Table 2). Most GPs (90%) worked in private general practice; most (65%) worked part-time. Most responding GPs (74%) obtained their primary medical degree in Australia or New Zealand; 35% spoke a second language. Forty percent of GPs reported that fewer than 5% of their clients were from hepatitis B endemic countries. Only four respondents (3%) reported being a prescriber of CHB treatment. (Table 2).

**Table 2:** Demographic characteristics of responded GPs of the survey

Characteristics	n (%) (*N* = 134)
**Female**	71 (53)
**Median age (IQR)**	56 (47–64)
**Type of practice** ^**a**^	
Private general practice	120 (90)
Aboriginal health service	11 (8)
Community health general practice	5 (4)
Other	4 (3)
**Working hour per week (median, IQR)**	32 (24–40)
**Working hours**	
Part time (< 40 h/week)	87 (65)
Full time (> = 40 h/week)	47 (35)
**CHB prevalence of participant’s practice PHN**	
0.5%- 1%	81 (60)
1.0- 1.7%	53 (40)
**Country where primary medical degree was obtained**	
Australia/ New Zealand	99 (74)
Overseas than Australia/New Zealand	35 (26)
**Speaks a language other than English with patients**	
No	84 (65)
Yes	47 (35)
Top five languages spoken other than English: Mandarin, Cantonese, Italian, French, Hindi	
**Patient profile: estimated proportion of clients born in hepatitis B endemic countries (outlined in a map)**	
< 5%	54 (40)
5–24%	50 (37)
> 25%	19 (14)
Unsure	11 (8)
**Hepatitis B Sect. 100 community prescriber**	
Yes	4 (3)
No or Unsure	125 (90)
Not reported	5 (7)

*CHB* Chronic hepatitis B., *HBV* Hepatitis B virus, *IQR* Inter-quartile range, *PHN* Primary health network

^a^multiple choices allowed

### Knowledge, attitudes, and confidence in hepatitis B practice

Overall, respondents demonstrated good knowledge in identifying populations at increased risk of hepatitis B infection with 94–99% correctly identifying at-risk populations including gay, bisexual, men who have sex with men, people who inject drugs, people living with HIV and/or hepatitis C infection, sex workers, and close contacts of people with CHB (Table 3). However only 79% and 87%, respectively, indicated that they would screen for hepatitis B in people from culturally and linguistically diverse communities or Aboriginal and Torres Strait Islander people (Table 3). Eighty-two percent of participants correctly interpreted active hepatitis B serology; ninety percent were aware of treatments available for CHB. In terms of the timing of treatment initiation, only 23% correctly identify the statement “*treatment can be initiated at any phase of hepatitis B infection*” as inaccurate in accordance with current guidelines [23, 24].

**Table 3:** Knowledge, perceived GPs role, attitudes, perceived barriers and facilitators related to hepatitis B practice among survey respondents

Items	n (%) (*N* = 134)
**Knowledge and awareness**	**Participants answering correctly (n, %)**
I would screen hepatitis B for the following population groups:	
CALD communities, particularly if born overseas (true)	106 (79)
Gay, bisexual and other MSM (true)	129 (96)
People who inject drugs (true)	133 (99)
Aboriginal and Torres Strait Islander people (true)	117 (87)
Close contacts of people with hepatitis B (true)	126 (94)
People with HIV and/or hepatitis C (true)	132 (99)
Sex workers (true)	129 (96)
Chronic hepatitis B infection is a major cause of hepatocellular carcinoma (HCC) in Australia	120 (90)
Accurate interpretation of “HBsAg positive, anti-HBc positive, anti-HBs negative” (active hepatitis B infection (chronic or acute))	110 (82)
Patients with active viral replication and active liver damage should be considered for treatment (true)	129 (96)
Treatment is available for hepatitis B (true)	120 (90)
Treatment can be initiated at any phase of hepatitis B infection (false)	30 (23) ^a^
Aware that hepatitis B medications could be dispensed in the community (yes)	51 (40)
**Attitudes and perceived GPs roles in providing CHB-related care**	**Participants agreeing the statement (n, %)**
It’s part of my work as a GP:	
Screening for HBV in patients with increased risk	127 (95)
Monitoring chronic hepatitis B	115 (86)
Prescribing HBV medication for eligible patients	39 (29)
Screening for HCC	105 (78)
None of above	2 (1)
**Intentions to become a hepatitis B prescriber (of those who are not or unsure, ** ***n*** ** = 123)**	
Yes	29 (24)
No	44 (35)
Unsure	50 (41)
**Belief and confidence** (on a scale of 0 to 10 where 10 means strongly agree/very confident/ important)	**Median score, IQR ** ^b^
Agreement level of the statement “it will benefit public health if I test for HBV among my high-risk patients”	10 (9–10)
Agreement level of the statement “it will benefit public health if I monitor chronic hepatitis B for my patients, regardless of specialists’ input”	8 (7–10)
Confidence level to monitor chronic hepatitis B	7 (5–8.5)
Confidence level to initiate treatment for hepatitis B	3 (2–5)
Importance level of “screen and manage chronic hepatitis B” compared to other priorities in practice	8 (7–10)
**Perceived barriers to providing hepatitis B testing or management**	**Participants agreeing the statement (n, %)**
Lack of time	51 (38)
Unclear guidelines	39 (29)
Lack of reminders	27 (20)
Lack of financial incentive	13 (10)
The difficulty of initiating the conversation	10 (7)
**Perceived facilitators to providing hepatitis B testing or mangement**	**Participants agreeing the statement (n, %)**
Clear guidelines on best practice would be a facilitator	96 (72)
Continuing medical education would be a facilitator	95 (71)
Online resources would be a facilitator	54 (40)
An education resource on plain language for my patients would be a facilitator	50 (37)
Medicare rebate would be a facilitator	44 (33)
Encouragement from colleagues would be a facilitator	24 (18)

^a ^*n* = 133, ^b ^*n* = 131

Most participants agreed that it is part of their GP role to test hepatitis B for patients with increased risk (95%), monitor CHB (86%) and screen for hepatocellular carcinoma (HCC) (78%). However, only 29% agreed that one of their responsibilities was to prescribe CHB treatment for their patients, and of the 123 non-S100 prescribers, 76% indicated no intention of becoming or were unsure whether they would become S100 prescribers.

Respondents reported being generally confident in monitoring CHB (with or without treatment), scoring a median confidence level of 7 (out of 10), but less confident in initiating treatment, with a median confidence score of 3 (out of 10).

### Perceived barriers and facilitators related to hepatitis B practice

Of the barriers and facilitators to providing CHB-related care listed in the questionnaire, the most frequently identified barrier was lack of time (38%), and the most frequently identified facilitator was clear guidelines on best practice (72%) (Table 3). Thirty-nine participants provided free-text feedback on barriers and 10 participants provided comments on support needs; categorised details and representative quotes are presented in Table 4.

**Table 4:** Self-reported barriers and support needs to providing hepatitis B-related care (categorised free-text comments)

Barriers	Support needs
• Patient-related factors - *“Very low risk in my patient population”* - *“Transient patients”* - *“Patient occasionally decline testing”* • Knowledge and confidence - *“Lack of knowledge”* - *“Screening is easy but I know a lot less about management”* • Competing priorities and lack of time - *“Other much more common diseases to screen”* - *“We are overloaded with multiple comorbidities with our complex patients. We can’t do everything as GPs on the rebates we get and the lack of time”* • Geographic barriers in accessing healthcare facilities - *“500 km to closest place to get ultrasound* + *no fibroscan here”*	• Innovative/ guideline to incorporating hepatitis B testing into routine clinical care * - “Usual (HBV) screening packaged as with chronic disease screening or for STI”* • Continued education - *“Need further education”* • Specialists’ support - *“Availability of shared care with a specialist”* • Education resources and programs targeting the community - *“Patient resources in Burmese languages”* - *“Community information program targeting at-risk groups”*

## Discussion

The study examined hepatitis B-related knowledge, attitudes, barriers and support needs of Australian GPs in providing CHB-related care. The study found most respondents agreed that hepatitis B testing, monitoring, and HCC surveillance were part of their work as a GP, and reported a reasonable level of expertise and confidence in delivering these services. However, most GPs did not see prescribing CHB treatment as part of their professional role. There was a knowledge gap about treatment initiation among responding GPs, and a lack of confidence in treating CHB. Several barriers to providing CHB-related care were identified, including patient-related factors, operational factors in general practice, and individual-level barriers for GPs, including lack of time, competing priorities, and a lack of knowledge and confidence. Support needs suggested by surveyed GPs included clear guidelines, further training, testing package integrated with other diseases screening, patient education resources, and specialist’ support.

Our study found that overall, GP respondents exhibited accurate knowledge about hepatitis B testing, but there was a trend that fewer GPs knew populations at greatest risk of CHB infection in Australia, namely people from culturally and linguistically diverse communities, particularly if born overseas, and Aboriginal and Torres Strait Islander people. Most GPs acknowledged that testing and monitoring for CHB is a component of their job, and showed moderate confidence in providing these services, again was consistent with previous surveys [25].

In contrast to their knowledge and attitudes with respect to providing testing and monitoring for CHB, responding GPs showed low levels of knowledge and confidence about treatment, consistent with previous research [26, 27]. Furthermore, this study found that participants felt that CHB treatment prescribing was not part of their role, with only a minority intending to become a prescriber. This highlights a need to raise awareness among GPs or consider treatment incentives [10, 28].

Barriers to delivering CHB-related care in primary care were identified at multiple levels in this study including client-related factors (e.g. refusal of testing), health system-related factors (e.g. health services accessibility in remote areas), and individual-level factors (such as competing priorities, and lack of knowledge and support).

While the most frequent chosen facilitator was having clear guidelines, several resources and tools are available to guide best practice in CHB clinical management, but GPs are reported to rarely use them [7]. Potential solutions to implement clinical guidelines in primary care include education, reminders, audits and feedback, and provider incentives [29]. In addition to interventions targeting GPs, a recent Australian study found CHB management in primary care settings being supported at an organisation level with a multidisciplinary team (e.g. practice nurses and practice managers involved in case finding and patient engagement), practice tools (such as a recall system) and clinical management support (e.g. supports from specialists, shared care, a clear referral protocol, or a computerised decision support system) [8, 9, 30–33]. A model of care with support from specialists was found to be highly acceptable among GPs [25]; a shared care model, (that is, suitable patients with CHB could be referred from specialists back to GPs and receive ongoing management at primary care with specialists support), was found to be beneficial in improving confidence and capacity building among primary care providers [2, 34].

This study has several limitations. The response rate was low compared to many other surveys of GPs, despite the short questionnaire, choice of completion methods and two follow up reminders [35]. Possible reasons are a lack of interest in CHB, or the time and resource commitment required to complete the questionnaire [36]. The low response rate means our findings of knowledge, confidence or attitudes towards providing CHB-related care from respondents may not be representative, and the results should be interpreted with caution. In particular, it is possible that GPs who responded had a relatively high interest in CHB and hence greater knowledge of testing and management than non-responding GPs and Australian GPs on average. We acknowledge that there might be different interpretation of some questions, such as the question asking which group the participant would screen for hepatitis B infection, while the aim was to understand participants’ awareness of at-risk population, it might be interpreted as a question reflecting daily practice among participants. The question with the statement that “*treatment can be initiated at any phase of hepatitis B infection*” (which deemed as an incorrect statement in the survey), was an over-simplification of hepatitis B treatment initiation. While antiviral therapy is generally reserved for certain phases of CHB, technically, it can be indicated in certain conditions (such as cirrhosis), regardless of CHB phases. Thus, this result may be biased due to inconsistent interpretation of the statement, and future survey needs to be cautious of oversimplifying clinical circumstances.

In summary, this study found that while most surveyed GPs had a good understanding of hepatitis B testing, most reported a lack of understanding, expertise, confidence, and intention to administer medication for CHB. The findings of this work highlight the need to engage GPs in providing CHB-related care and generate interest through skill-building interventions. Ongoing research is required to measure the impact of interventions that aim to increase GP engagement in CHB care.

## Supplementary Information

**Additional file 1.**

## Data Availability

The datasets generated during and/or analysed during the current study are not publicly available due to ethics requirement but are available from the corresponding author on reasonable request.

## Abbreviations

AMPCo: Australian Medical Publishing Company
CHB: Chronic hepatitis B
GP: General practitioner
HCC: Hepatocellular Carcinoma
IQRs: Interquartile ranges
PHN: Primary Health Network
