# Supplementary material for: A survey of knowledge, attitudes, barriers and support needs in providing hepatitis B care among GPs practising in Australia
Source: BMC Prim Care. 2022 Jun 2;23:137. doi: 10.1186/s12875-022-01754-3 (PMC9161590; doi:10.1186/s12875-022-01754-3)
Supplement: Supplementary file 1 — Additional file 1. [file 12875_2022_1754_MOESM1_ESM.docx]

## Supplementary tables

Table s1. Questionnaire “Hepatitis B management by general practitioners”

**Part I. Demographics**

1. What is your gender?

- Male
- Female

1. What year were you born?

__­­­­­

1. At what type of general practice do you work?

- Private general practice
- Aboriginal health service
- Community health general practice
- Other. Please specify________________________________________

1. How many clinical hours do you work each week?
2. What is the postcode of your practice?

__­­­­­

(If you practise in multiple clinics, select the postcode of the clinic in which you practise most frequently)

1. In which country did you complete your primary medical degree?

- Australia
- Outside Australia. Please specify: ________________

1. Do you speak a language other than English with your patients, when required?

- Yes. Please specify_____________
- No

| 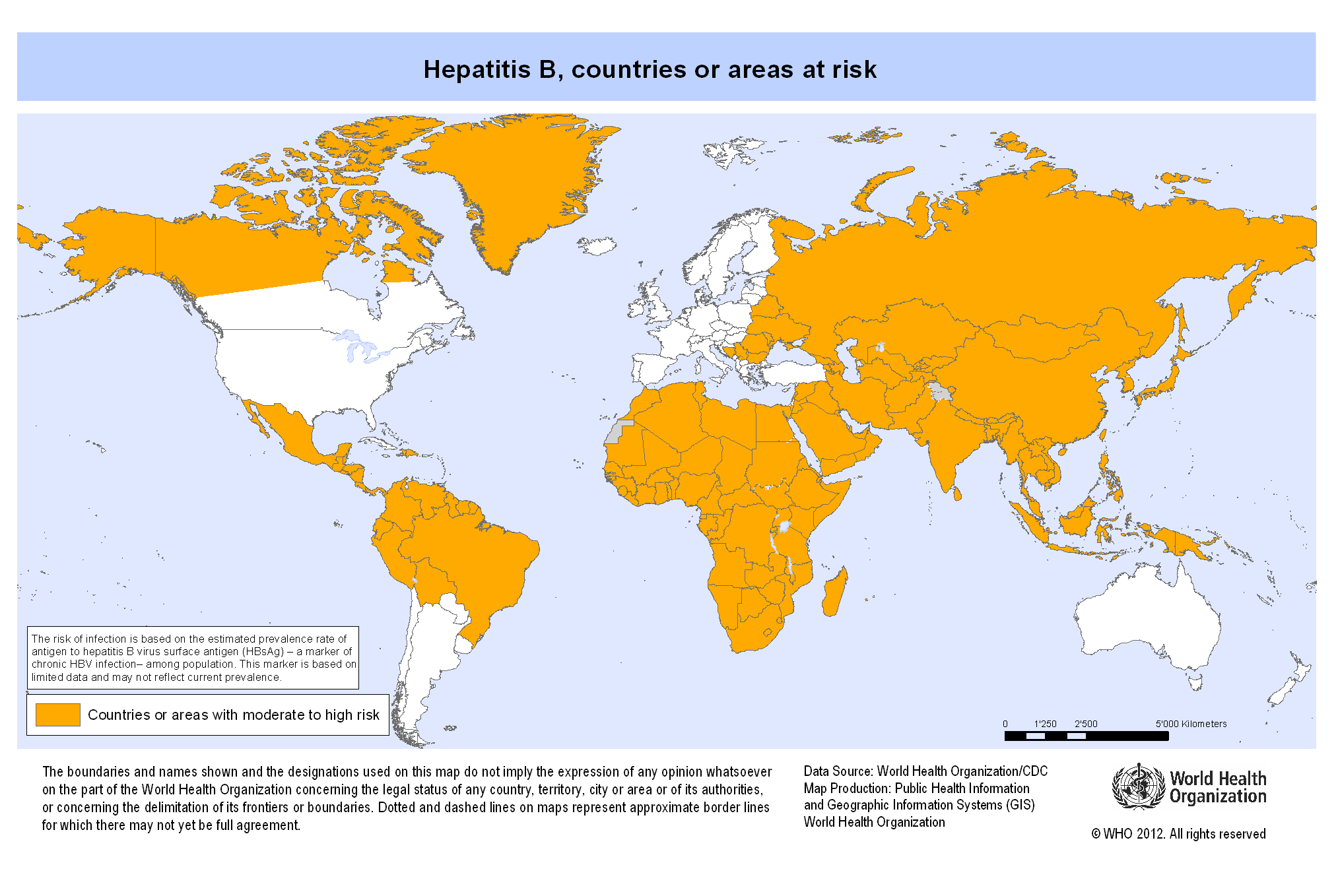 |
| --- |
| 1. Looking at the map above, what proportion of your patients do you estimate were born in a hepatitis B endemic country (countries shaded in orange)?  - 0 - <5% - 5–24% - 25–49% - 50–74% - >75% - Unsure |

**Part II.** **The following questions apply to your knowledge and current practice with respect to hepatitis B management.**

1. Which of the following population groups would you screen for hepatitis B infection? **(Select all that apply)**

- Culturally and linguistically diverse communities, particularly if born overseas
- Gay, bisexual and other men who have sex with men
- People who inject drugs
- Aboriginal and Torres Strait Islander people
- Close contacts of people who have hepatitis B
- People with HIV and/or hepatitis C
- Sex workers
- Sexually active people
- Don’t know

1. How would you interpret the following serology?

| HBsAg | positive |
| --- | --- |
| anti-HBc | positive |
| anti-HBs | negative |

- Active hepatitis B infection (chronic or acute)
- Vaccinated against hepatitis B infection
- Susceptible to hepatitis B infection
- Resolved HBV infection
- Unsure

| 1. Please indicate your response to the following statements by crossing the box (X): | | | |
| --- | --- | --- | --- |
|  | True | False | Unsure |
| 1. Chronic hepatitis B infection is a major cause of hepatocellular carcinoma (HCC) in Australia | □ | □ | □ |
| 1. Patients with active viral replication and active liver damage should be considered for treatment | □ | □ | □ |
| 1. Treatment is available for hepatitis B | □ | □ | □ |
| 1. Treatment can be initiated at any phase of hepatitis B infection | □ | □ | □ |

1. **In the last two years**, who is primarily responsible for HCC screening for your patients with chronic hepatitis B?

- Myself
- Other GPs at my practice
- Specialists via referral
- Shared care of specialists and GPs
- Other. Please specify____________________________________________

**Part III.** **The following questions ask about your preferences and beliefs with respect to the management of hepatitis B.**

1. Which of the following statements do you believe is part of your work as a GP? (Select all that apply)

- Screening for HBV in my patients with increased risk
- Monitoring chronic hepatitis B for my patients
- Prescribing HBV medication for my patients who are eligible
- Screening for HCC for my patients living with chronic hepatitis B
- None of the above

1. How strongly do you agree or disagree with the following statements? (On a scale of 1 to 10, with 1 being “strongly disagree” and 10 being “strongly agree”)
2. **I am confident I could initiate treatment for my patients living with hepatitis B if clinically indicated, based on my knowledge.**

| Strongly disagree | |  |  |  |  |  |  | Strongly agree | |
| --- | --- | --- | --- | --- | --- | --- | --- | --- | --- |
| 1 | 2 | 3 | 4 | 5 | 6 | 7 | 8 | 9 | 10 |

1. **I am confident in monitoring chronic hepatitis B for my patients as necessary.**

| Strongly disagree | |  |  |  |  |  |  | Strongly agree | |
| --- | --- | --- | --- | --- | --- | --- | --- | --- | --- |
| 1 | 2 | 3 | 4 | 5 | 6 | 7 | 8 | 9 | 10 |

1. **It will benefit public health if I test for HBV among my high-risk patients.**

| Strongly disagree | |  |  |  |  |  |  | Strongly agree | |
| --- | --- | --- | --- | --- | --- | --- | --- | --- | --- |
| 1 | 2 | 3 | 4 | 5 | 6 | 7 | 8 | 9 | 10 |

1. **It will benefit public health if I monitor chronic hepatitis B for my patients, regardless of specialists’ input.**

| Strongly disagree | |  |  |  |  |  |  | Strongly agree | |
| --- | --- | --- | --- | --- | --- | --- | --- | --- | --- |
| 1 | 2 | 3 | 4 | 5 | 6 | 7 | 8 | 9 | 10 |

1. Considering your other priorities in your practice, how important do you think it is for you to screen and manage chronic hepatitis B? (On a scale of 1 to 10, with 1 being “Not important at all” and 10 being “Very important”)

| Not important at all | | |  |  |  |  |  | Very important | |
| --- | --- | --- | --- | --- | --- | --- | --- | --- | --- |
| 1 | 2 | 3 | 4 | 5 | 6 | 7 | 8 | 9 | 10 |

**Part IV.** **The following questions help us understand the barriers and facilitators for your hepatitis B management.**

1. Which guidelines or resources, if any, have you used for **the management of** **hepatitis B** within the **previous 2 years?** (Select all that apply)

- None
- Red Book (*Guidelines for Preventive Activities in General Practice*, RACGP)
- Decision making in hepatitis B (ASHM)
- *B Positive – all you wanted to know about hepatitis B – a guide for primary care providers* (ASHM)
- “Hep B Help” website
- Health pathway (if available through PHN)
- National testing policy
- Other. Please specify_______________________________________________

1. Which of the factors below prevent you from effectively screening for HBV or management of hepatitis B? (Select all that apply)

- Not enough time
- Lack of reminders
- Unclear guidelines
- Difficulty in initiating the conversation with my patients
- Lack of financial incentive
- Other. Please specify___________

1. Which of the factors below would facilitate better screening for HBV or management of hepatitis B? (Select all that apply)

- Clear guidelines on best practice
- Continuing medical education
- Online resources
- An education resource on plain language for my patients
- Encouragement from colleagues
- Medicare rebate
- Other. Please specify___________________________________________________

1. Are you aware that from 1 July 2015, hepatitis B medications could be dispensed in the community?

- Yes
- No
- Unsure

1. Are you an accredited s100 hepatitis B Community Prescriber?

- Yes **You’ve finished the survey**.
- No
- Unsure

1. Would you like to become a hepatitis B s100 Community Prescriber?

- Yes
- No
- Unsure

1. If you would like more information about how to become a hepatitis B s100 Community Prescriber, please provide an email address or postal address:_________________________

Table s2. Critical appraisal checklist for a questionnaire study

| Number | Description of the standard | Brief description of how the standard has been met | Page number |
| --- | --- | --- | --- |
|  | **Research question and study design** |  |  |
| 1 | What information did the researchers seek to obtain? | Knowledge, attitudes, perceived barriers and facilitators among GPs in providing hepatitis B-related care | 7 |
| 2 | Was a questionnaire the most appropriate method and if not, what design might have been more appropriate? | Yes | 7 |
| 3 | Were there any existing measures (questionnaires) that the researchers could have used? If so, why was a new one developed and was this justified? | No existing validated instruments to measure knowledge, attitudes, perceived barriers and facilitators towards deliering hepatitis B-related care | / |
| 4 | Were the views of consumers sought about the design, distribution, and administration of the questionnaire? | Yes, study design was guided by advisory committee where GPs were an important part; 5 GPs were consulted to test advise on the survey design. | 8 |
|  | **Validity and reliability** |  |  |
| 5 | What claims for validity have been made, and are they justified? (In other words, what evidence is there that the instrument measures what it sets out to measure?) | The instrument was developed to be used in the context of surveying Australian GPs; it was developed with the guidance of advisory committee which consist of specialists (infectious diseases, hepatology), GPs, nurses, epidemiologists, health promotion program managers, and public health experts. | 8-9 |
| 6 | What claims for reliability have been made, and are they justified? (In other words, what evidence is there that the instrument provides stable responses over time and between researchers?) | The questionnaire was delivered in a written form with clearly stated questions; the questionnaire did not contain questions seeking participants’ reporting their practice behaviour. | 8-9 |
|  | **Format** | |  |
| 7 | Was the title of the questionnaire appropriate and if not, what were its limitations? | The questionnaire titled “hepatitis B management by general practitioners” in short; it’s concise and relevant to the purpose of the study. | Table s1 |
| 8 | What format did the questionnaire take, and were open and closed questions used appropriately? | The questionnaire has a combined question formats including tick boxes, rating scale, close-ended questions and open-ended questions with free text comment options. | 8-9; Table s1 |
| 9 | Were easy, non-threatening questions placed at the beginning of the measure and sensitive ones near the end? | Questions are generally easy and short. The beginning section asks participants basic demographic information. | 8-9; Table s1 |
| 10 | Was the questionnaire kept as brief as the study allowed? | Yes, the 21-item questionnaire was short and concise. | 8-9; Table s1 |
| 11 | Did the questions make sense, and could the participants in the sample understand them? Were any questions ambiguous or overly complicated? | The questions were easy to understand, survey length was short and clear. It was tested within 5 GPs and all commented being easy to understand, no question raised about the clarity of the questions. | 8-9; Table s1 |
| Instructions | |  |  |
| 12 | Did the questionnaire contain adequate instructions for completion—eg example answers, or an explanation of whether a ticked or written response was required? | The questionnaire did not contain an demonstration question; however instructions were given where relevant, such as “select all that apply” or “select…by crossing the circle” | Table s1 |
| 13 | Were participants told how to return the questionnaire once completed? | Yes, a cover letter with the information of how to completed and return the questionnaire was included. | 7 |
| 14 | Did the questionnaire contain an explanation of the research, a summary of what would happen to the data, and a thank you message? | Yes, a cover letter and a participant information sheet were provided all participants together with the paper questionnaire. | 7 |
| Piloting | |  |  |
| 15 | Was the questionnaire adequately piloted in terms of the method and means of administration, on people who were representative of the study population? | The questionnaire was piloted among 5 GPs mainly on the contents. The methods of delivery was guided by study advisory committee and the consultation with AMPCo who provided the contact details of the surveyed GPs. | 7 |
| 16 | How was the piloting exercise undertaken—what details are given? | Piloting was completed with volunteer GPs referred by community partners (CCV) and research team members. The study purpose and questionnaire was provided to volunteer GPs individually via email, who were asked to provide comments on questionnaire clarity, validity, relevance, if any missed answers, language, format, and other suggestions. | 8 |
| 17 | In what ways was the definitive instrument changed as a result of piloting? | Answers to specific questions were amended as a result of piloting, such as adding an answer option of “all of the above” to question 9. | Table s1 |
| Sampling | |  |  |
| 18 | What was the sampling frame for the definitive study and was it sufficiently large and representative? | Random sample from the largest commercial database of medical professionals. Sample size was calculated based on GP population, margin of error and potential response rate. | 7 |
| 19 | Was the instrument suitable for all participants and potential participants? In particular, did it take account of the likely range of physical/mental/cognitive abilities, language/literacy, understanding of numbers/scaling, and perceived threat of questions or questioner? | Yes, we believe the instrument is suitable for all participants. | / |
| Distribution, administration and response | | |  |
| 20 | How was the questionnaire distributed? | The questionnaire was posted to potential participants. | 7 |
| 21 | How was the questionnaire administered? | Participants were requested to complete the paper survey by themselves or online survey with a linked provided. | 7 |
| 22 | Were the response rates reported fully, including details of participants who were unsuitable for the research or refused to take part? | Yes. | 10; Figure 1 |
| 23 | Have any potential response biases been discussed? | Yes. | 10-11; Table 1 |
| Coding and analysis | |  |  |
| 24 | What sort of analysis was carried out and was this appropriate? (eg correct statistical tests for quantitative answers, qualitative analysis for open ended questions) | Please see methods- data analysis. | 9-10 |
| 25 | What measures were in place to maintain the accuracy of the data, and were these adequate? | All data were entered into REDCap and reviewed; raw data were then exported for analysis using Stata. | 9 |
| 26 | Is there any evidence of data dredging—that is, analyses that were not hypothesis driven? | Data were mainly descriptively presented. | / |
| Results | |  |  |
| 27 | What were the results and were all relevant data reported? | Yes, relevant results were reported in the results section. Two questions (q12 and q16) asked about participants practice behaviour and was not part of the scope of the presenting paper, thus the findings were included in supplementary material table s3. | 10-15 |
| 28 | Are quantitative results definitive (significant), and are relevant non-significant results also reported? | Only relevant results were reported | 10-15 |
| 29 | Have qualitative results been adequately interpreted (e.g. using an explicit theoretical framework), and have any quotes been properly justified and contextualised? | Qualitative data (that is, free-text comments) were coded and themes were summarised. | 9 |
| Conclusions and discussion | |  |  |
| 30 | What do the results mean and have the researchers drawn an appropriate link between the data and their conclusions? | This was reported in the discussion section. | 16-19 |
| 31 | Have the findings been placed within the wider body of knowledge in the field (eg via a comprehensive literature review), and are any recommendations justified? | This was reported in the discussion section. | 16-19 |

Table s3. Results of survey questions not reported in this paper.

| Items | n(%) (N=134) |
| --- | --- |
| In the last two years, who is primarily responsible for HCC screening for your patients with chronic hepatitis B: |  |
| Myself | 63 (47) |
| Other GPs at my practice | 8 (6) |
| Specialists via referral | 19 (14) |
| Shared care of specialists and GPs | 41 (31) |
| Other | 8 (6) |
| Which guidelines or resources, if any, have you used for the management of hepatitis B within the previous 2 years: |  |
| None | 61 (46) |
| Red Book (Guidelines for Preventive Activities in General Practice, RACGP) | 26 (19) |
| Decision making in hepatitis B (ASHM) | 26 (19) |
| B Positive – all you wanted to know about hepatitis B – a guide for primary care providers (ASHM) | 20 (15) |
| “Hep B Help” website | 9 (7) |
| Health pathway (if available through PHN) | 14 (10) |
| National testing policy | 15 (11) |
| Other | 14 (10) |
